# Proteomic Analysis of Spatial Heterogeneity Identifies HMGB2 as Putative Biomarker of Tumor Progression in Adult-Type Diffuse Astrocytomas

**DOI:** 10.3390/cancers16081516

**Published:** 2024-04-16

**Authors:** Aline P. Becker, Valesio Becker, Joseph McElroy, Amy Webb, Chunhua Han, Yingshi Guo, Erica H. Bell, Jessica Fleming, Ilinca Popp, Ori Staszewski, Marco Prinz, Jose J. Otero, Saikh Jaharul Haque, Anca-Ligia Grosu, Arnab Chakravarti

**Affiliations:** 1 Department of Radiation Oncology, The Ohio State University, Columbus, OH 43210, USA; aline.becker@osumc.edu (A.P.B.); valesio.becker@osumc.edu (V.B.); chunhua.han@osumc.edu (C.H.); yingshi.guo@osumc.edu (Y.G.); jessica.fleming@osumc.edu (J.F.); jahar.haque@osumc.edu (S.J.H.); 2 Center for Biostatistics, The Ohio State University, Columbus, OH 43210, USA; joseph.mcelroy@osumc.edu; 3 School of Biomedical Science-Biomedical Informatics, The Ohio State University, Columbus, OH 43210, USA; amy.hite@osumc.edu; 4 Department of Neurology, The Ohio State University, Columbus, OH 43210, USA; erica.bell@osumc.edu; 5 Department of Radiation Oncology, University of Freiburg, 79110 Freiburg, Germany; ilinca.popp@uniklinik-freiburg.de (I.P.); anca.grosu@uniklinik-freiburg.de (A.-L.G.); 6 Institute of Neuropathology, Medical Faculty of the Saarland University, 66421 Homburg, Germany; ori.staszewski@uks.eu; 7 Institute of Neuropathology, Faculty of Medicine, University of Freiburg, 79106 Freiburg, Germany; marco.prinz@uniklinik-freiburg.de; 8 Signalling Research Centres BIOSS & CIBSS, University of Freiburg, 79098 Freiburg, Germany; 9 Department of Pathology, The Ohio State University, Columbus, OH 43210, USA; jose.otero@osumc.edu

**Keywords:** tumor heterogeneity, mass spectrometry, proteomic profile, HMGB2, glioma, glioblastoma, gene methylation

## Abstract

**Simple Summary:**

Adult-type astrocytomas usually present heterogeneous aspects under the microscope, reflecting their progression over time from grade 2 or 3 to grade 4, the highest possible grade. We identified a number of proteins and molecular pathways dysregulated in the high-grade areas of astrocytomas, which are involved in tumor evolution that could be targeted to avoid or detain glioma progression to higher grades. We identified HMGB2 as a potential biomarker of glioma evolution and predictive of response to treatment in more than 300 adult-type gliomas, using molecular profiling and immunohistochemistry, which are highly accessible for most pathology laboratories. HMGB2 expression increased even before histological markers of evolution appeared in grades 2 and 3 astrocytomas, and it was associated with poor survival. In glioblastomas, high HMGB2 expression identified tumors with better response to the standard treatment and could be used as additional inclusion/exclusion criterion to enroll patients in future clinical trials of new treatments.

**Abstract:**

Although grading is defined by the highest histological grade observed in a glioma, most high-grade gliomas retain areas with histology reminiscent of their low-grade counterparts. We sought to achieve the following: (i) identify proteins and molecular pathways involved in glioma evolution; and (ii) validate the high mobility group protein B2 (HMGB2) as a key player in tumor progression and as a prognostic/predictive biomarker for diffuse astrocytomas. We performed liquid chromatography tandem mass spectrometry (LC-MS/MS) in multiple areas of adult-type astrocytomas and validated our finding in multiplatform-omics studies and high-throughput IHC analysis. LC-MS/MSdetected proteomic signatures characterizing glioma evolution towards higher grades associated with, but not completely dependent, on IDH status. Spatial heterogeneity of diffuse astrocytomas was associated with dysregulation of specific molecular pathways, and HMGB2 was identified as a putative driver of tumor progression, and an early marker of worse overall survival in grades 2 and 3 diffuse gliomas, at least in part regulated by DNA methylation. In grade 4 astrocytomas, HMGB2 expression was strongly associated with proliferative activity and microvascular proliferation. Grounded in proteomic findings, our results showed that HMGB2 expression assessed by IHC detected early signs of tumor progression in grades 2 and 3 astrocytomas, as well as identified GBMs that had a better response to the standard chemoradiation with temozolomide.

## 1. Introduction

One of the main challenges in the management of adult-type gliomas is intratumor heterogeneity, which plays important roles in tumor diagnosis, progression, and resistance to treatment. Intratumor heterogeneity is driven by tumor cell clones and subclones, as well as by components of the tumor microenvironment (TME), such as blood vessels and immune cells (reviewed in [1]). In the diagnostic routine of diffuse gliomas, histological grading is defined by the area with the highest histological grade, based on the hallmarks of nuclear atypia, proliferative activity, microvascular proliferation, and necrosis [2]. However, up to 60% of glioblastomas (GBMs) and astrocytomas grade 4 IDH mutant (A4 IDHmut) retain areas with histological features reminiscent of their lower-grade counterparts [3,4]. The variance in signal transduction pathway activation between these morphologically diverse areas remains to be extensively studied and may provide instructive information on tumor progression. Transcriptomic studies usually yield classifiers accounting for thousands of genes [5], but although protein-coding genes correspond to less than 2% of the human genome, the protein expression profile is more directly related to the cellular phenotype than high-throughput gene expression analysis. Therefore, we hypothesized that mass spectrometry-based proteomic analysis may improve the detection and understanding of spatial and temporal heterogeneity in adult-type diffuse gliomas.

Recent advances in protein chemistry have diversified the biomarker discovery toolbox in translational oncology. Specifically, the application of liquid chromatography tandem MS (LC-MS/MS) to archival pathology tissue that has undergone harsh fixation and preservation through the formalin fixation/paraffin embedding (FFPE) process now enables us to directly identify proteins that can be further validated as novel biomarkers, with results similar to the ones obtained with frozen tissue [6,7]. In addition, compared to other methods of proteomics analyses, such as matrix-assisted laser desorption/ionization time-of-flight mass spectrometry (MALDI-TOF MS), which is a fingerprinting-based method, LC-MS/MS allows for the analyses of amino acid sequence of proteins (top-down and middle-down proteomics) and peptides (bottom-up proteomics), post-translational modifications, and protein–protein interaction (reviewed in [8]), with increased sensitivity and reliability. The application of LC-MS/MS in the elucidation of complex interactions in systems biology is instrumental in the discovery of new biomarkers of disease that can be clinically validated with routine methods, such as immunohistochemistry (IHC).

Given the potential for discovery of biomarkers and actionable targets leveraging the proteomic data, we set out to identify regional proteomic signatures with LC-MS/MS in diffuse gliomas samples selected based on histopathological criteria, aiming to achieve the following: (i) identify proteins and molecular pathways involved in glioma evolution; and (ii) validate the high mobility group protein B2 (HMGB2) as a key player in tumor progression and as a prognostic/predictive biomarker for diffuse astrocytomas.

## 2. Materials and Methods

### 2.1. Discovery Cohort

**Figure 1:**
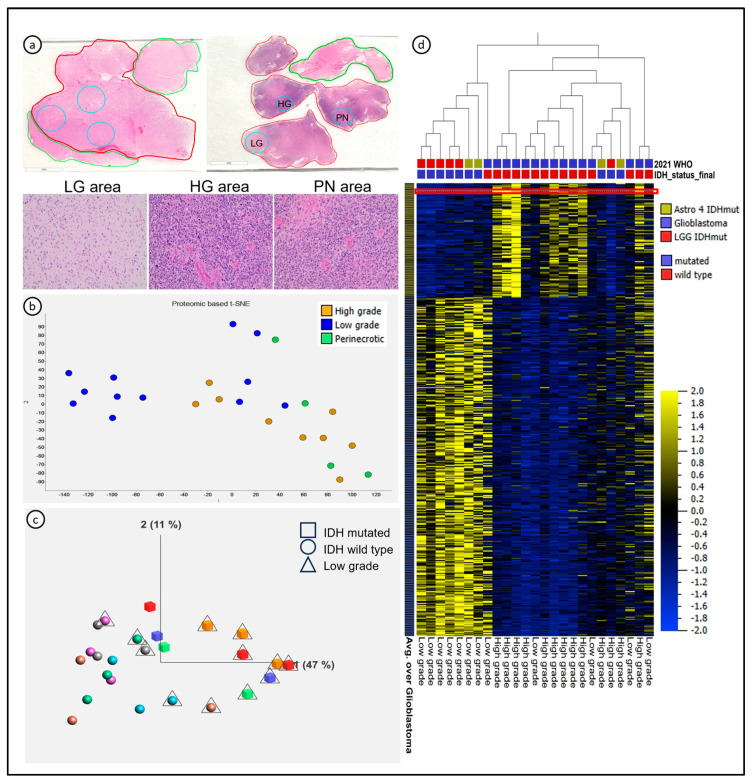
Spatial heterogeneity of diffuse astrocytomas characterized with mass spectrometry-based proteomics. (**a**) Representative cases with areas collected from single (top left) and multiple fragments (top right) in the paraffin block. Histological areas representative of low-grade (LG) area, high-grade (HG) area, and peri-necrotic (PN) area in the bottom row were collected from the case with multiple fragments. (**b**) t-Distributed Stochastic Neighbor Embedding (t-SNE) showed clusters of samples identifying LG and HG areas; the PN areas clustered with the HG areas. (**c**) Principal Component Analysis showed clusters according to low-grade (triangles) and high-grade (no additional marker) histological features—each color represents one patient in the study (N = 9). (**d**) Heatmap revealed clusters of LG and HG samples based on proteomic signatures; the red line identifies *HMGB2* expression as one of the top 10 proteins more expressed in HG areas of diffuse astrocytomas.

We performed LC-MS/MS in multiple samples from nine adult-type diffuse gliomas (Table 1) resected at the University of Freiburg and analyzed at the Ohio State University (OSU), under the oversight of the Institutional Review Board (IRB) 2013C0020. Hematoxylin and eosin (H&E)-stained slides were evaluated by an experienced neuropathologist (A.P.B.), who selected the regions of interest (ROIs) according to histological features of low-grade, high-grade, and peri-necrotic areas (PN). Histologically, low-grade was defined as areas with low cellularity and low mitotic activity, and the absence of microvascular proliferation; high-grade was defined as areas of high cellularity, high mitotic activity, and the presence of microvascular proliferation; PN areas were defined as areas of viable tumor around necrosis. A total of twenty-five FFPE tissue areas within distances ranging from 5 to 16 mm (average 9.6 mm) from each other in the paraffin block (Figure 1a and Supplementary Table S1) were included for analysis.

#### 2.1.1. Protein Isolation and Mass Spectrometry

Tissue cores of 1 mm diameter and no less than 1 mm thick were collected from the paraffin blocks using an AutoTiss EverBio^®^ tissue microarray (TMA) semi-automated platform (EverBio Technology Inc., New Taipei City, Taiwan), targeting the ROIs with at least 70% of tumor cells, and submitted to protein extraction at OSU, following our previously published protocol [9,10,11]. Briefly, the tissue was deparaffinized using heptane, followed by dehydration with methanol. Proteins were extracted from the resulting pellet by incubation in an extraction buffer at 100 °C for 20 min, followed by a 2 h incubation at 80 °C, and recovered by centrifugation [12] using Qproteome FFPE Tissue Kit (Qiagen Inc., Germantown, MD, USA).

Eight micrograms (8 µg) of protein were processed at Case Western Reserve University Center for Proteomics with Dual Digest of LysC/Tryspin, as previously reported by our group [9,10,11]. The minimum protein concentration required for analysis is 180 ng/µL. All our samples reached at least that minimum amount. A pooled sample was created from aliquots of the samples. All samples were analyzed with a 4 h liquid chromatography tandem MS (LC-MS/MS) for label-free expression proteomics. Data were processed in Peaks Software—http://www.bioinfor.com/peaks-studio/ (accessed on 11 October 2023), v. 8.5 patch with the quantification provided at the protein level.

#### 2.1.2. Pathway Analysis

Pathway analysis to identify differences in dysregulated pathways in low-grade versus high-grade areas of the adult-type diffuse gliomas was performed by inputting the protein-coding genes associated with the proteins differentially expressed. We first performed a “Core Analysis” to identify the enrichment of canonical pathways and/or possible biases, as the analysis was limited to the protein-coding genes. We input the whole list of detected proteins for the background analysis using http://metascape.org, accessed on 11 October 2023. Then, we input the “significant” list of proteins for foreground analysis (meaning those with differential expression between low-grade areas and high-grade areas). We then looked for enrichment of pathways in the foreground. Pathways with a z score > +2 were considered “up regulated” and those with a z score < −2 were regarded as “down regulated”. The analysis was performed with Metascape (http://metascape.org, accessed on 11 October 2023) [13] for Gene Ontology enrichment analysis, protein–protein interaction, and metanalysis of high-grade and low-grade areas; and with Ingenuity Pathway Analysis (IPA) software v. 23.0 (QIAGEN Inc., https://www.qiagenbioinformatics.com/products/ingenuity-pathway-analysis, accessed on 6 November 2023). After selecting our target (HMGB2), we also validated it in public databases (www.wikipathways.org and https://glioblastoma.alleninstitute.org/, accessed on 13 November 2023).

### 2.2. Validation Cohorts

#### 2.2.1. Molecular Characterization (Validation Cohort 1)

FFPE tissue from 63 diffuse gliomas from the University of Freiburg (validation cohort 1—Supplementary Table S2) originally diagnosed as histological grades 2 and 3 gliomas, was processed and analyzed as previously published by our group [9,14,15] for genomic, epigenomic, and transcriptomic profiling, updating the original diagnoses to the 2021 WHO classification of adult-type gliomas [16].

- Genomic and methylomic profiling:

Briefly, DNA was extracted from FFPE tissues using a combination of Recoverall Total Nucleic Acids Isolation (ThermoFisher Scientific, Waltham, MA, USA) and Epicentre Masterpure DNA Purification (Illumina, San Diego, CA, USA) kits. DNA quantity and quality were assessed using the Qubit dsDNA High Sensitivity Assay Kit (ThermoFisher Scientific, Waltham, MA, USA). Approximately 250 ng of DNA was used for a customized Ion AmpliSeq (Thermo Fisher Scientific, Waltham, MA, USA) targeted DNA panel sequencing. Sequence alignment and variant calling were performed using the Ion Suite and Reporter software v. 5.16 to assess for noncanonical *IDH1/2* mutations and *EGFR* mutations. *TERT* mutations were determined with Sanger sequencing. Codeletion of chromosomes 1p and 19q, homozygous deletion of *CDKN2A*/*B*, as well as chromosome 7 gains and chromosome 10 losses were determined by Affymetrix Oncoscan FFPE Assay and/or Illumina HumanMethylation450BeadChip (Illumina 450k array) [14]. Global methylation profiling of the tumors was performed with 250 ng of DNA at the University of Southern California Epigenome Center (Los Angeles, CA, USA) with an Illumina 450K array according to our previous protocols [9]. Data were processed using the R package “minfi” with the hg19 annotation. Data were Noob normalized and M-value [17] transformed.

- Transcriptomic profiling:

Total RNA was extracted from FFPE glioma tissues with the RNAeasy kit (Qiagen Inc., Germantown, MD, USA). Purity assessment was performed with Nanodrop spectrophotometer (Thermo Fisher Scientific, Waltham, MA, USA) to select RNAs with a 260/280 ratio of at least 1.8 for the microarray analysis. cRNA was hybridized to the Affymetrix HTA ClariomD Array (Thermo Fisher Scientific, Waltham, MA, USA). Normalization (SST-RMA) and summarization were carried out using Affymetrix transcriptome analysis console software v.4.0.2.

Molecular data publicly available from the Cancer Genome Atlas Program (TCGA) via CBio Portal (cBioPortal for Cancer Genomics), from the Chinese Glioma Genome Atlas (CGGA) (Home | CGGA—Chinese Glioma Genome Atlas), and from the Ivy Glioblastoma Atlas Project (Home :: Ivy Glioblastoma Atlas Project (https://alleninstitute.org, accessed on 13 November 2023)) were used for validation of our findings [18,19,20,21].

#### 2.2.2. Histopathology and Immunohistochemistry (IHC) (Validation Cohort 2)

For the clinical validation of HMGB2 assessment, we performed IHC. Staining for HMGB2 expression assessment was performed in Dako Link 48 automatic stainer, with antigen retrieval at low pH in a steamer (Dako S1699), followed by a peroxidase block for 5 min, incubation with the primary antibody HMGB2 (Invitrogen, MA5-36118) 1:1000 dilution for 30 min, Labelled Polymer Flex/HRP for 30 min, Flex DAB substrate (Dako Envision Flex Kit) for 10 min, and counter staining with hematoxylin. The HMGB2 IHC slides were scored semi-quantitatively for the percentage of positive cells multiplied by the intensity of reaction (0—negative, 1—weak, 2—moderate, 3—strong), which resulted in a HMGB2 score ranging from 0 to 285. The upper quartile (percentile 75—p75 = 108) score was used as a cut-off value for “high” versus “low” HMGB2 expression, as it represents roughly 50% of tumor cells with moderate intensity or 33% of tumor cells with strong intensity.

In addition to validation cohort 1 (diffuse gliomas histological grades 2 and 3), we analyzed 336 grade 4 astrocytomas (validation cohort 2), both IDH wild type glioblastoma (GBM) and astrocytoma IDH-mutant WHO grade 4 (A4 IDHmut) [22], obtained from the Department of Pathology at OSU with complete clinical data. These samples were arranged in 12 TMAs, representing multiple ROIs from each tumor. Histological markers of gliomas grading (cellularity, mitotic activity, microvascular proliferation, percentage of necrosis) were assessed in the H&E stain of validation cohort 2. Assessment of HMGB2 expression and of IDH status in validation cohort 2 was performed with IHC, using the protocol described above, and with the antibody anti-*IDH1* R132H H09 (Dianova, New York, NY, USA, 1:800) [23], using the Leica Bond III stainer and Leica Bond Refine DAB kits, as per manufacturer’s protocol, respectively.

### 2.3. Biostatistical Analyses

The results of the LC-MS/MS were analyzed using statistical software Qlucore Omics Explorer 3.8 (Qlucore, New York, NY, USA), using t-Distributed Stochastic Neighbor Embedding (t-SNE), Principal component Analysis (PCA), and heatmap with hierarchical clustering to identify clusters of samples with similar profiles. Validation and correlation studies were performed with Python and IBM SPSS Statistics, v 29.0.1. The association between *HMGB2* expression and methylation was determined using a linear model with expression as the outcome and a main effect of methylation (separately for each probe). Cox proportional hazards was employed for identifying continuous expression and methylation associations with overall survival (OS) in univariable models. For Kaplan–Meier plots, expression and methylation were median or p75 dichotomized, and associations were tested with the log-rank test.

## 3. Results

### 3.1. LC-MS/MS-Detected Proteomic Signatures Characterizing Glioma Evolution towards Higher Grades, Which Were Associated with, but Not Completely Dependent on, IDH Status

Due to the known spatial heterogeneity of diffusely infiltrating gliomas, we set out to identify regional proteomic signatures using histological criteria. Low-grade, high-grade, and peri-necrotic (PN) areas were designated by a trained neuropathologist, and the ROIs were micro-dissected for proteomic analysis of the FFPE tissue samples (Figure 1a). These procedures quantified a total of 9222 peptides, mapped to 2655 non-redundant proteins, and 2622 protein-coding genes, identifying two clusters. The samples labeled as PN clustered very closely with the high-grade samples (Figure 1b,c); therefore, we carried out a further analysis, differentiating low-grade areas (LG, n = 12 samples), characterized by low cell density and inconspicuous nuclear atypia, from high-grade areas (HG, including both HG and PN, n = 13 samples), characterized by high nuclear density and atypia, proliferative activity, and microvascular proliferation.

We first investigated differences according to the IDH status, and overall, 631 proteins were differentially expressed in IDHwt compared with IDHmut astrocytomas (*p* = 0.03, FDR = 0.1). Then, we sought to identify differences between histological grades and noted 424 proteins with differential expression between HG and LG areas (*p* < 0.03, FDR < 0.1), where 103 proteins were overexpressed in the HG cluster, and 317 proteins were overexpressed in the LG cluster (Figure 1d). In total, 294 proteins differentially expressed according to histological grades overlapped with the proteins differentially expressed according to the IDH status.

While the LG proteomic signature was mostly observed in IDHmut astrocytomas, the HG cluster contained both IDHwt and IDHmut astrocytomas. The color code on Figure 1c depicts the internal differences between HG and LG areas of single tumors. The LG areas of IDHwt astrocytomas clustered close to the HG areas of IDHmut astrocytomas. The HG areas of IDHmut astrocytomas presented a loss of expression of most proteins related to the LG proteomic signature, suggesting that the proteomic signature characterizing glioma evolution towards higher grades is associated with, but not completely dependent on, IDH status (Figure 1c,d). We conclude that LC-MS/MS was successful in detecting intratumor regional differences in FFPE tissue based on histopathological features associated with glioma grade.

### 3.2. Spatial Heterogeneity of Diffuse Astrocytomas Was Associated with Dysregulation of Specific Molecular Pathways and HMGB2 Was Identified as a Putative Driver of Tumor Progression

To test the hypothesis that the proteomic signatures associated with HG versus LG areas in astrocytomas reflected different biological processes underlying glioma evolution occurred simultaneously in the tumor, we performed pathway analysis by inputting the protein-coding genes associated with the proteins detected in the LC-MS/MS.

**Figure 2:**
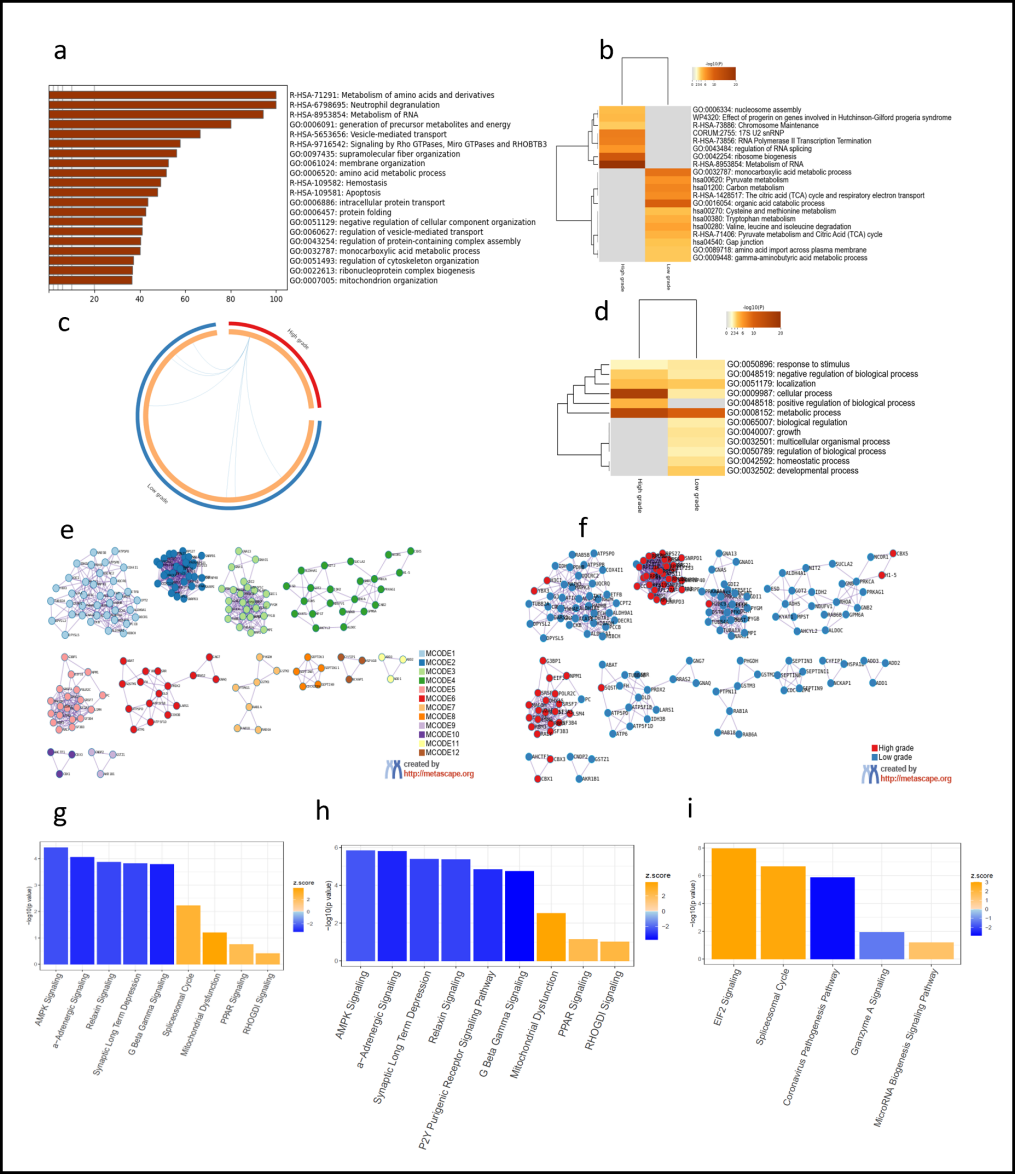
Gene Ontology (GO) and protein–protein interaction analysis confirmed the regional heterogeneity of diffuse astrocytomas associated with histopathological features. (**a**) Background GO analysis of protein-coding genes associated with abundant proteins in diffuse gliomas showed an unbiased dataset. (**b**) Metanalysis showed different enrichment patterns in HG and LG areas. (**c**) In the Circos plot, genes lists do not overlap (external arcs represent the identity of each gene list); however, some different genes fall under the same ontology term (shown by the blue lines). (**d**) Heatmap specifying the overlap of ontology terms observed in the Circos plot. (**e**) Protein–protein interactions identified 12 MCODE components. (**f**) MCODE2, MCODE5, and MCODE10 (R-HSA-8953854—metabolism of RNA; hsa03040—spliceosome; GO:0006325—chromatin organization) are enriched in the HG areas. (**g**,**h**) IPA pathway analysis showed the same patterns of GO, with background (**g**) and LG areas (**h**) showing a similar enrichment pattern. (**i**) Canonical pathways de-regulated in HG areas were mainly related to enhanced mRNA and protein synthesis and inhibition of apoptosis.

To reduce biases related to our targeted approach, as our dataset consisted of only of fragments detectable by the proteomic analysis, we performed protein background analysis by inputting the complete list of genes (n = 2622) associated with the proteins expressed in the cohort for gene ontology (GO) analysis (Figure 2a, Supplementary Table S3). Our results showed minimal differences, whether including the protein panel as a background or including all known proteins as the background.

We then input the top 400 protein-coding genes correspondent to proteins differentially expressed in HG versus LG areas (*p* = 0.03, FDR = 0.1) for metanalysis (Supplementary Table S4) and observed that the enrichment in LG areas was reminiscent of the background analysis (Figure 2a,b), centered in energy metabolism and amino acid metabolism, while HG areas showed an enrichment of processes associated with cell proliferation (Figure 2b). There were few overlaps between the HG and LG areas (Figure 2c,d), including GO:0008152 (metabolic process). Protein–protein interactions showed enrichment of three specific MCODE nodes (R-HSA-8953854—metabolism of RNA; hsa03040—spliceosome; GO:0006325—chromatin organization) in the HG areas, compared with LG areas (Figure 2e,f). The full MCODE analysis is available in Supplementary Table S5.

IPA Core background analysis confirmed the unbiased results observed in GO and showed dysregulation of over 200 canonical pathways (Supplementary Table S6). Overall, for both the HG and LG areas, AMPK-signaling and synaptic long-term depression pathways were the most significantly downregulated pathways, while PPAR signaling, mitochondrial dysfunction, spliceosomal pathways were up-regulated (Figure 2g). Similar to the GO analysis, the canonical pathways specifically up-regulated in LG areas were similar to the background analysis (Figure 2h, Supplementary Table S7).

HG areas demonstrated upregulation of pathways related to protein and mRNA synthesis (EIF2 signaling and spliceosome cycle, respectively), which seemed to be driven by abundant ribosomal proteins, and downregulation of the apoptotic pathway granzyme A signaling. The granzyme A signaling pathway consists of five protein-coding genes: *ANP32A*, *H1-5*, *HMGB2*, *LMNB1*, and *SET* (Figure 2i) (Supplementary Table S8). Not surprisingly, HMGB2 was one of the top 10 proteins differentially overexpressed in HG areas versus LG areas of diffuse gliomas (*p* = 0.003, FDR = 0.099) (Figure 1c).

Here, we showed that histopathological intratumor heterogeneity is associated with dysregulation of specific molecular pathways. Upregulation of anti-apoptotic and cell proliferation pathways was observed in the HG areas in diffuse gliomas, and HMGB2 was identified as a potential driver of glioma progression.

*HMGB2* was reported to be part of the canonical pathway Retinoblastoma gene in cancer (WP2446) [24,25], recently incorporated in the p21-p53-RB pathway [26], and is involved in DNA double-strand break repair Therefore, it contributes to resistance to the standard treatment of gliomas with radiation (RT) [27] and temozolomide (TMZ) [28]. This justified further analyses of *HMGB2* expression in diffuse astrocytomas.

### 3.3. High HMGB2 Expression, Both at mRNA and Protein Levels, Is an Early Marker of Worse Overall Survival in Grades 2 and 3 Diffuse Gliomas, and It Is, at Least in Part, Regulated by DNA Methylation

We then sought to investigate the relationship between *HMGB2* mRNA expression with glioma grade, possible underlying genomic and/or epigenomic regulatory mechanisms, and patient survival using public datasets. This was followed by validation in an institutional cohort (validation cohort 1), which was composed of grades 2 and 3 gliomas devoid of necrosis or microvascular proliferation (Supplementary Table S2). We reclassified the TCGA dataset using molecular data from Ceccarelli et al. [29] and the validation cohort 1 according to the 2021 WHO classification, accounting for IDH and 1p19q status and molecular alterations in chromosomes 7/10, *TERT*, *EGFR,* and/or *CDKN2A/B* [16]. We performed molecular characterization of validation cohort 1 with an Ion AmpliSeq (Thermo Fisher Scientific, Waltham, MA, USA) customized DNA panel sequencing, Sanger sequencing, and Affymetrix Oncoscan, according to our previously published protocols [14].

**Figure 3:**
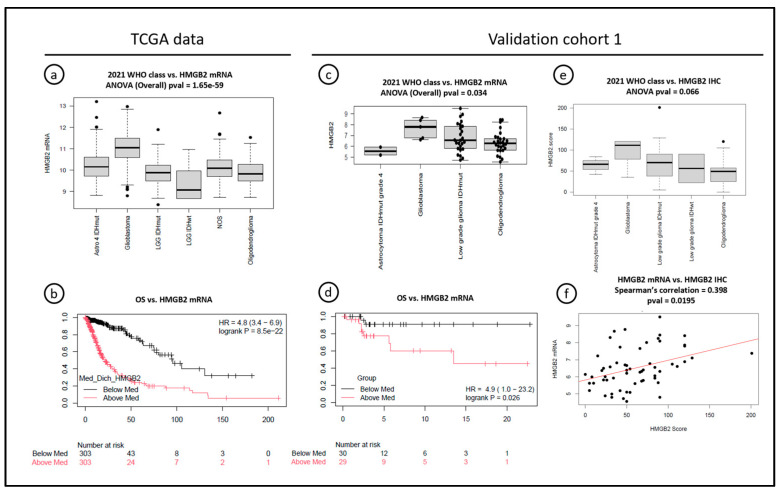
Associations between *HMGB2* mRNA expression, overall survival, and HMGB2 expression, assessed with immunohistochemistry. (**a**,**b**) TCGA data: *HMGB2* mRNA expression is increased in GBMs compared to other 2021 WHO diagnoses and associated with poor overall survival in diffuse gliomas. (**c**,**d**) Validation cohort 1: *HMGB2* mRNA expression is increased in molecular GBMs compared to other 2021 WHO diagnoses in histologically grades 2 and 3 gliomas, and associated with worse overall survival in this cohort. (**e**,**f**) Validation cohort 1: HMGB2 protein expression (immunohistochemistry) is marginally increased in molecular GBMs compared to other 2021 WHO diagnoses in the same cohort, and mRNA results were moderately associated with the HMGB2 score at IHC. Note: “Low-grade glioma IDHwt” shown in the plot 2021 WHO class vs. HMGB2 IHC is currently diagnosed as Glioma, NEC under the 2021 WHO classification.

In public databases, rare (0.39%) gene mutations were observed in *HMGB2* [18,19,20,30], and only 2% of the cases presented gene amplification (N = 1) or deep deletion (N = 14), all but one observed in the TCGA dataset labeled “lower grade gliomas” [18,19]. Nonetheless, *HMGB2* mRNA expression significantly increased with glioma grade (mRNASeq_325 dataset—ANOVA *p* = 3 × 10^−25^) in CGGA [20]. In TCGA (N = 669), *HMGB2* mRNA expression was higher in tumors from the “Glioblastoma multiforme” dataset than in the “Brain Lower Grade Glioma” (T-test *p* = 9.79 × 10^−45^—Supplementary Figure S1), and in GBMs compared with other diagnoses, after reclassification according to 2021 WHO (*p* = 1.65 × 10^−59^). This reclassification was performed by assessing *CDKN2A/B* and *EGFR* status in the original cases from Ceccarelli et al. (2016) [29] (Figure 3a, Supplementary Table S9, adapted from Ceccarelli et al., 2016 [29]).

Concerning the relationship between *HMGB2* mRNA expression and overall survival, TCGA data showed that *HMGB2* mRNA above average was strongly related to worse OS when including all histological grades (Figure 3b). However, CGGA data showed that high *HMGB2* mRNA expression was significantly related to OS in grades 2 and 3 diffuse gliomas (*p* = 0.039 and *p* < 0.0001), but not in GBMs (grade 4—*p* = 0.25).

Given that our discovery cohort (n = 9) was not powered for survival studies, and considering the conflicting results of the public datasets, *HMGB2* mRNA expression was assessed using the Affymetrix HTA ClariomD array and compared with survival data from validation cohort 1. The reclassification was achieved in 60 out of 63 grades 2 and 3 gliomas of that cohort (95%) (Supplementary Figure S2). We observed that 2/60 (3%) IDHmut astrocytomas were reclassified as A4IDHmut due to *CDKN2A/B* homozygous deletion and 5/60 (8%) of IDHwt grades 2 and 3 were reclassified as GBMs (grade 4) due to one or more alterations in *TERT*, *EGFR*, and chromosomes 7 and 10, under the 2021 WHO criteria. Those molecular GBMs had *HMGB2* mRNA expression significantly higher than the other confirmed grades 2 and 3 astrocytomas and oligodendrogliomas (Figure 3c), including “low-grade IDHwt astrocytomas” without other molecular alterations, which would now be classified as glioma, NEC. The low number of A4 IDHmut prevented a conclusion about the association of *HMGB2* expression with IDH status. High *HMGB2* mRNA expression was significantly associated with worse OS in grades 2 and 3 diffuse gliomas (*p* = 0.026—Figure 3d).

Since rare genomic alterations have been reported in *HMGB2*, we investigated if DNA methylation could explain increased *HMGB2* mRNA expression in higher grade diffuse gliomas. We assessed DNA methylation in validation cohort 1 using the Illumina 450K array to identify probes associated with *HMGB2* mRNA expression and with OS. Out of twenty-five methylation probes located in the region of *HMGB2,* hypermethylation of six probes were strongly associated with reduced *HMGB2* expression; hypermethylation of three of those probes (cg1937134, cg21499459 and cg08269316), all located in the gene body, were also significantly associated with better OS (Supplementary Table S9 and Figure S3), substantiating that lower *HMGB2* expression is related to better survival in grades 2 and 3 gliomas.

Finally, because the correlation between mRNA expression and protein expression assessed by IHC is not always straightforward, we sought to evaluate if significant correlation could justify the use of IHC as a more available surrogate method for HMGB2 expression assessment. We compared mRNA data and IHC scores from 58 samples from validation cohort 1 and showed a moderate correlation between the methods (Spearman’s correlation = 0.398, *p* = 0.0195), We also confirmed marginally higher HMGB2 protein expression in the molecular GBMs, compared to other diagnoses in this cohort, although it was not statistically significant (*p* = 0.066) (Figure 3e,f).

Taken together, these results suggest that *HMGB2* expression is, at least in part, regulated by DNA methylation, and increases in grades 2 and 3 astrocytomas before the histological characteristics of grade 4 (necrosis and microvascular proliferation) are evident. More importantly, increased expression may be detected with IHC in the routine neuropathology laboratory. Therefore, HMGB2 may be an early predictor of a worse prognosis for grades 2 and 3 astrocytomas.

### 3.4. HMGB2 Is Significantly Associated with Histopathological Markers of Grade 4 Astrocytomas, and It Is Minimally Expressed in Non-Neoplastic Glial Cells

Public data showed that *HMGB2* mRNA expression was significantly higher in the “cellular tumor”, than in the surrounding infiltrative areas [21], and that proneural glioblastomas (which are usually associated with IDH mutations) [29,31] had higher *HMGB2* expression than mesenchymal and classical glioblastomas (Supplementary Figure S4). Nevertheless, the association of *HMGB2* expression with IDH status, and the relative expression in non-neoplastic tissue adjacent to an infiltrative glioma, remained elusive.

Since we observed a good reliability of IHC staining in grades 2 and 3 gliomas, here we sought to thoroughly characterize HMGB2 expression in validation cohort 2, a large cohort of grade 4 astrocytomas originally diagnosed as GBMs and arranged in 12 TMAs (Table 2). Our aim was to describe HMGB2 expression in tumor cells and in the non-neoplastic adjacent brain tissue, its subcellular location and relationship with histopathological hallmarks of tumor grade and IDH status. Longitudinal analysis was performed in a subset of recurrent tumors compared with their primary counterparts (n = 25 matched pairs). The tumors in which the LG and HG areas were observed in the same H&E-stained slide (N = 16 tumors) were used to evaluate spatial heterogeneity of HMGB2 expression. A subset of 165 H&E-stained slides were available for evaluation of histological features and association with HMGB2 expression.

**Figure 4:**
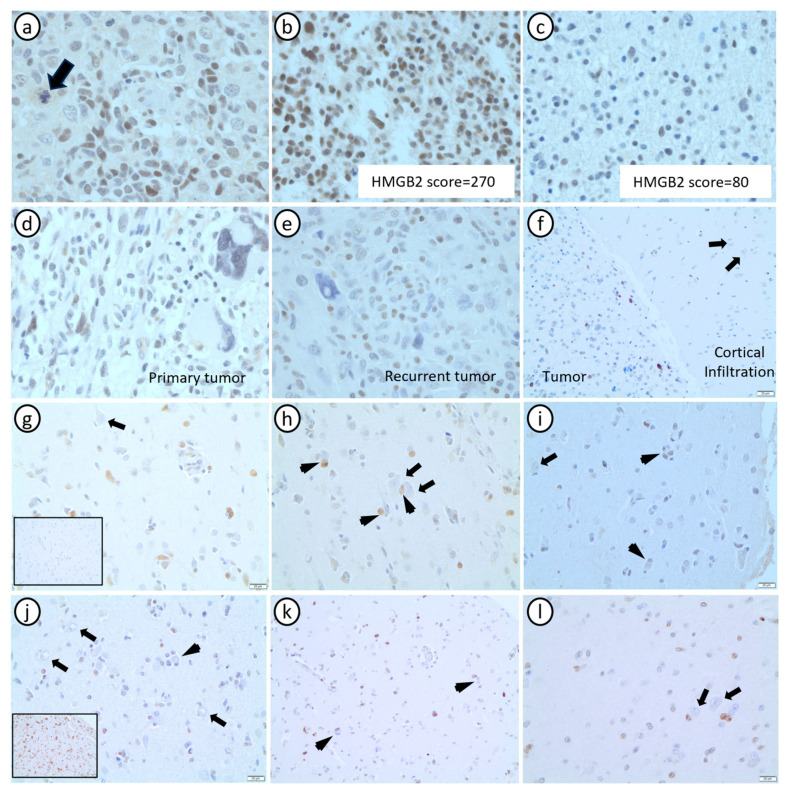
Histopathological characterization of HMGB2 expression in grade 4 astrocytomas. (**a**) In the tumor bulk, HMGB2 expression was expressed in the nuclei of tumor cells (arrow: atypical mitotic figure). (**b**,**c**) HMGB2 spatial intratumor heterogeneity is observed in high-grade area (**b**) and low-grade area (**c**) from the same GBM. (**d**,**e**) HMGB2 expression in primary (pre-treatment—(**d**)) and recurrent (post-treatment—(**e**)) GBMs from the same patient, showing reduced HMGB2 expression in giant multinucleated cells in both timepoints. (**f**) HMGB2 nuclear expression in scattered tumor cells in the tumor bulk and in the adjacent area of cortical infiltration, where the expression is negative in non-neoplastic cells. (**g**–**i**) Areas of cortical infiltration of 3 GBMs showing HMGB2 expression only in tumor cells, but not in non-neoplastic neurons and glial cells (inset 4g: IDH1 R132H negative). (**j**–**l**) Areas of cortical infiltration of an A4IDHmut (**j**) and two grade 2/3 astrocytomas IDHmut ((**k**,**l**)—confirmed with sequencing) showing HMGB2 expression only in tumor cells, but not in non-neoplastic neurons and glial cells (inset (**j**): IDH1 R132H diffusely positive). In (**f**–**l**), arrows point to neurons (HMGB2 negative) and arrowheads point to perineuronal satellitosis of HMGB2 positive tumor cells.

In tumor cells, HMGB2 expression was mainly nuclear (Figure 4a). Similar to our initial proteomic analysis, HMGB2 expression was slightly higher in the HG areas (mean score = 69.37) compared to the LG areas (mean score = 63.43) within single tumors (Figure 4b,c), but this difference did not reach statistical significance (*p* = 0.06). The HMGB2 score was marginally higher in A4 IDHmut (mean score = 94.13) than in GBMs (mean score = 69.67) (*p* = 0.056), and significantly higher in primary tumors (mean score = 74.72) compared to their paired recurrences (mean score = 41.84) (*p* = 0.027) (Figure 4b,c). There was no significant difference in HMGB2 expression between sex and age (*p* = 0.672 and *p* = 0.257, respectively).

Importantly, in the areas representative of the infiltrative edge, HMGB2 expression was sometimes cytoplasmic in tumor cells, while being mostly absent from non-neoplastic oligodendrocytes, astrocytes, and neurons (Figure 4f–l), both in GBMs and in grades 2 and 3 and A4 IDHmut. This finding corroborates data from public databases, such as The Human Protein Atlas (https://www.proteinatlas.org/, accessed on 13 November 2023) [32], which shows positive immunostaining in glial cells and neurons when using antibodies that detect other proteins of the HMGB family (HMGB1 and HMGB3) in the cerebellum and in the basal ganglia, but low or no expression when using a more specific antibody anti-HMGB2, similar to the antibody we used in this study. HMGB2 expression was reduced or absent in multinucleated giant tumor cells in both primary and recurrent tumors (Figure 4d,e).

Overall, young age (i.e., <60 years old) was strongly associated with IDH mutation (*p* = 4.8 × 10^−6^), male sex (*p* = 0.02), and longer OS (*p* = 6.0 × 10^−5^), confirming that our series is representative of the usual grade 4 astrocytoma epidemiology. While HMGB2 expression (continuous values) was not associated with any of the clinical features, it was significantly associated with histological criteria that define grade 4 gliomas, such as mitotic count (representing proliferative activity—*p* = 0.0092) and glomeruloid vessels (*p* = 0.017). Associations between HMGB expression and clinicopathological features are summarized in Supplementary Figure S5.

We confirmed that HMGB2 is a highly specific marker of diffuse gliomas, compared to non-neoplastic brain tissue. Consistent with our results from pathway analysis, IHC confirmed a strong association with cell proliferation. The change in HMGB2 subcellular expression in tumor cells from the bulk of the tumor, compared to the infiltrative border, remains to be elucidated and may be related to changes in HMGB2 function.

### 3.5. High HMGB2 Expression Identifies Glioblastomas with Better Response to Treatment

While high HMGB2 expression was related to worse survival in grades 2 and 3 diffuse gliomas, this was not confirmed by public data for GBMs (*p* = 0.25) [20]. To evaluate the prognostic potential of HMGB2 expression in grade 4 astrocytomas, we performed univariable and multivariable analyses in our validation cohort 2 to assess associations with OS. For these calculations, the upper quartile (percentile 75—p75 = 108) score was used as cut-off value for “high” versus “low” HMGB2 expression, as it represents roughly 50% of tumor cells with moderate intensity or 33% of tumor cells with strong intensity.

**Figure 5:**
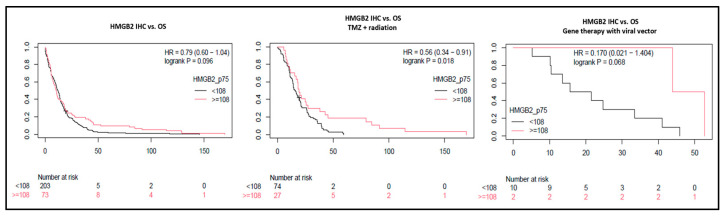
HMGB2 effects on overall survival in grade 4 glioma patients. In GBM and A4 IDHmut, overall survival difference was not significant according to HMGB2 expression at IHC. However, high HMGB2 expression identified patients with better response to treatment and longer OS after radiation plus temozolomide and after gene therapy with an adenoviral vector.

Contrary to our results from transcriptomics in grade 2/3 astrocytomas, in univariable analysis, the difference in survival according to higher or lower HMGB2 expression (mean survival: 15 × 23 months; 95% CI: 13.1–17.9 × 15.4–30.7 months, respectively) was not significant (*p* = 0.096—Figure 5), corroborating results from public databases [20,33]. Nevertheless, in multivariable analysis including both clinical and histopathological variables, age, use of any adjuvant treatment, mitotic activity, and high HMGB2 expression were significantly associated with OS (Table 3). The lack of difference in survival according to IDH mutations in this cohort may be due to underestimation of the full range of IDH mutations with the use of the specific antibody.

This finding raised the question of whether HMGB2 expression would affect the response to the main treatment modalities. Therefore, we calculated OS stratified by adjuvant treatment and observed that patients treated with RT + TMZ had OS significantly longer when HMGB2 expression was high (*p* = 0.018—Figure 5). Nevertheless, the difference was not significant compared to the HMGB2 expression in patients who did not receive adjuvant treatment (*p* = 0.8) or received only RT (*p* = 0.62) after surgery. In a small subset of patients treated with adenoviral vector injection gene therapy (NCT00589875) [34], patients with higher HMGB2 expression had better long-term response (mean OS = 48.3 months) than patients with low expression (mean OS = 22.6 months); however, this difference was not significant (*p* = 0.068, Figure 5). Taken together, these results suggest that while HMGB2 may play a role in the progression of grades 2 and 3 diffuse gliomas, in fully developed grade 4 diffuse astrocytomas, higher HMGB2 expression may be a biomarker of treatment response.

## 4. Discussion

In IDH mutant astrocytomas, progression from grades 2 to 4, previously known as secondary GBM, is deemed the natural course of the disease [35]. For IDHwt astrocytomas, although tumor initiation events can occur many years before a GBM is diagnosed [36], the current WHO classification no longer recognizes the diagnosis of IDHwt low-grade gliomas [16]. Nevertheless, areas resembling low-grade gliomas are frequently observed at histopathology in grade 4 astrocytomas of any IDH status [3,4]. While spatial differences between the cellular tumor (bulk) and its infiltrative margins have been previously described [21], the comparison between LG and HG areas coexisting at short distances within IDHwt and IDHmut diffuse astrocytomas may shed light on the pathobiology of glioma evolution and identify actionable molecular targets to avoid or detain glioma progression to higher grades.

Our evaluation of diffuse astrocytomas (1p19q intact gliomas) grades 2–4 with LC-MS/MS showed not only unique proteomic signatures according to histological grade within a single tumor, but also associations of those proteins with different hallmarks of cancer [37,38]. Upregulation of pathways related to aberrant cell energy and metabolism (“Deregulating cell metabolism” [38]) observed in LG areas may simply reflect the enrichment of LG areas with IDHmut tumors in our study, as 2-HG also reduces the efficiency of cell metabolism [39]. On the contrary, HG areas derived from both IDHmut and IDHwt astrocytomas showed dysregulation of pathways related to “Sustaining proliferative signaling/Evading growth suppressors” and “Resisting cell death” [38], as suggested by the upregulation of pathways related to mRNA and protein synthesis (EIF2 and spliceosomal cycle pathways) and the downregulation of the apoptotic granzyme A pathway. We identified *HMGB2*, a component of both the granzyme A pathway and of the p21-p53-RB pathway, as one of the most abundant proteins in HG areas.

HMGB2 is a non-histone protein, part of the high-mobility group (HMG) superfamily [40], reported to participate in DNA-binding, chromatin remodeling, and DNA repair [40,41], therefore preventing apoptosis [42], probably through transactivation of the *BAX* gene promoter [43]. Although HMGB2 is the only member of the HMGB family with increased expression in gliomas compared to normal brain tissue and associated with survival in grades 2 and 3 gliomas [33], in GBMs, this association is controversial. Therefore, there is a knowledge gap about the role of HMGB2 in glioma progression and treatment response.

Here, we performed an extensive multi-omics study to discover and validate of HMGB2 as an early diagnostic and/or predictive marker of glioma progression and demonstrated one possible regulatory epigenetic mechanism of gene expression. Increased *HMGB2* expression with grade in gliomas [28,33,44] probably reflects the loss of cell differentiation and enhanced proliferative activity during glioma progression [2,45,46]. Here, we showed that this is an early event, occurring in GBMs originally diagnosed as grade 2 or 3 gliomas, prior to the histological characteristics of GBM becoming evident. While increased expression of HMGB2 is not tied to gene mutations and CNAs that rarely affect the gene, epigenetic mechanisms, such as previously reported micro-RNAs, were reported to regulate HMGB2 expression [47]. To our knowledge, we have shown for the first time that DNA methylation of CpG islands located at the body of *HMGB2* (cg19371349, cg21499459, cg08269316) also, at least in part, regulates HMGB2 expression and is strongly associated with OS in grades 2 and 3 gliomas.

To develop the clinical assessment of HMGB2, we used IHC to confirm the LC-MS/MS findings from our discovery cohort. We then demonstrated spatial heterogeneity in HMGB2 expression in a large cohort of GBMs and A4IDHmut. While the lack of molecular data from validation cohort 2 reduced our numbers for methylomic and transcriptomic correlations, the number of patients in that larger cohort enabled the survival analysis, including correlation with response to treatment and with specific histological features, such as proliferative activity in the tumors and HMGB2 expression in non-neoplastic cells. We therefore believe that both cohorts are complementary in the clinical information they provide. Besides detecting differences between LG and HG areas within the same tumor in grade 4 astrocytomas of validation cohort 2, we reported two major findings: (1) reduced HMGB2 expression in non-neoplastic glial cells and neurons, as previously reported [21,33,40,48]; and (2) a change in the subcellular HMGB2 expression from nuclear to cytoplasmic in infiltrating tumor cells. We hypothesize that this change probably reflects modifications in HMGB2 functions, as the nuclear expression observed in virtually all tumors is more related to DNA bending and gene transcription, while cytoplasmic expression has been related to prevention of apoptosis and enhanced immune response [42,49,50,51]. Additional studies are needed to prove this hypothesis.

The association of HMGB2 with IDH status is still elusive, and several knowledge gaps remain to be addressed. The difference in HMGB2 expression between IDHwt and IDHmut gliomas was not consistent across our cohorts and public data [20]. In grades 2 and 3 gliomas, IDH mutations seemed to be related to lower HMGB2 expression; however, in grade 4 astrocytomas, HMGB2 expression was significantly higher in A4IDHmut, which bear *CDKN2A/B* homozygous deletions [22,52], compared to GBMs at IHC. This supports the previously reported, yet insufficiently explored participation of *HMGB2* in the p21-p53-RB pathway [24,25].

Interestingly, contrary to previous studies showing that high HMGB2 expression was related to treatment resistance in vitro [27,28], here, we observed significantly better survival in patients with grade 4 diffuse astrocytomas (GBM and A4 IDHmut) treated with adjuvant chemoradiation. Several factors can explain this discrepancy, such as post-translational modifications [53], which may affect negatively HMGB2 function in vivo; and the significant correlation observed between high HMGB2 expression and proliferative activity may explain the better response to RT due to maximum radiation sensitivity being noted at G2 and M phases of the cell cycle [54]. More importantly, in vitro studies did not account for interactions with components of the TME. HMGB proteins (specifically HMGB1) are involved in the response to immunogenic cell death [55,56] occurring after RT, and HMGB2 is a known regulator of CD8+ T cells, required for anti-melanoma response [57]. Based on similarities between HMGB1 and HMGB2, we hypothesize that astrocytomas with high HMGB2 expression have a more powerful immune response to damage-associated molecular patterns (DAMPs), arising after RT plus temozolomide and/or oncolytic viral vectors injection [58]. This hypothesis is strengthened by the observed translocation of HMGB2 expression from nucleus to cytoplasm in the tumor cells at the infiltrative border, which has been previously described as part of the response to immunogenic cell death, and as a predictive biomarker for colon cancer [50,51,56]. Mechanistic studies are warranted to elucidate the relationship between HMGB2 subcellular expression, immune response to DAMPs, and outcomes of treatment.

Finally, based on our findings, due to the high specificity of HMGB2 expression in tumor cells, but not in normal brain cells, the role of HMGB2 expression as prognostic and/or predictive biomarker is promising. Although the high correlation with proliferative activity raises the possibility of overlapping with other markers, such as Ki67 proliferative index (PI), HMGB2 has some advantages over it. In addition to the overlapping between grades 3 and 4, which translates to a lack of specific cut-points for Ki67 PI [59], not even the use of automated digital analysis was able to establish an association between Ki67 PI and OS [60]. In contrast, we showed with multiple methods that increased HMGB2 expression identifies grades 2 and 3 gliomas with molecular alterations of GBM and A4 IDHmut (“molecular glioblastomas”) before the histological development of necrosis and microvascular proliferation takes place, and it is strongly associated with OS in public and institutional cohorts. Clinical validation of our results and of the IHC method for HMGB2 assessment in larger, independent cohorts and in CLIA-certified (or equivalent) facilities is needed to validate our findings and establish HMGB2 as prognostic biomarker of grades 2 and 3 astrocytoma evolution and predictive biomarker of response to treatment in grade 4 astrocytomas.

The main limitation of this retrospective study was the molecular profile being performed in FFPE samples. While the quality of the analyses may be subpar compared to analyses conducted on frozen tissue, two advantages arise: (1) reduction of noise in LC-MS/MS, as the abundant proteins detected will be more stable in the cell and resistant to fixative procedures; (2) improvement of the methods will enable studies of larger series of archive FFPE tissues, to overcome the limitations of the availability of frozen tissue. The second limitation was the lack of information about the *MGMT* and *TP53* status of the patients in the survival study (validation cohort 2), which could affect the therapeutic response and explain the conflicting survival results. Finally, the IDH status in validation cohort 2 was performed by IHC only, and the number of A4IDHmut may have been underestimated due to the lack of accounting for IDH mutations different from R132H. Therefore, conclusions regarding the relationship between HMGB2 and IDH status should be viewed with caution, and further validation is needed. Our future goals include performing mechanistic studies of HMGB2 functions using in vitro and in vivo models, including the elucidation of the crosstalk between HMGB2 expression in tumor cells and components of the TME in the immune response following immunogenic cell death induced by current treatment modalities.

## 5. Conclusions

Our approach of characterizing the proteomic profile of histopathology-targeted areas was successful in depicting temporo-spatial heterogeneity in diffuse astrocytomas and identified HMGB2 as a valuable, yet poorly explored, putative enabler of glioma progression. We validated our finding in multiplatform-omics studies and high-throughput IHC analysis, which raised several important hypotheses on the role of HMGB2 in astrocytoma progression and response to treatment, which are currently being investigated by our group.

Although LC-MS/MS proteomic studies in FFPE tissue are limited to abundant proteins resistant to the histological processing, rather than the exploration of the whole proteome, its effective role in discovering new actionable targets and developing more effective biomarkers is unparalleled. Grounded in proteomic findings, our results showed that HMGB2 expression assessed by IHC detected early signs of tumor progression in grades 2 and 3 astrocytomas, as well as identified GBMs and A4 IDHmut that had better response to the standard chemoradiation with temozolomide.

## Figures and Tables

**Table 1:** Characterization of the discovery cohort.

Patient	Sex	Age (Years)	Tumor Order	Original Diagnosis	2021 WHO	IDH Status	1p19q Status	OS (Months)	Samples per Block
1	M	28	P	AA	A3IDHmut	Mut	intact	31	3
2	F	24	P	DA	A2IDHmut	Mut	intact	2	3
3	F	49	R	GBM *	A4IDHmut	Mut	intact	15	2
4	F	55	R	GBM *	A4IDHmut	Mut	intact	16	2
5	M	86	P	GBM	GBM	WT	intact	6	3
6	F	56	P	GBM	GBM	WT	intact	22	3
7	M	81	P	GBM	GBM	WT	intact	10	3
8	M	69	P	GBM	GBM	WT	intact	4	3
9	F	44	R	GBM	GBM	WT	intact	6	3

M—male; F—female; P—primary; R—recurrent; AA—anaplastic astrocytoma, grade 3; DA—diffuse astrocytoma, grade 2; GBM *—GBM, IDHmut; GBM—glioblastoma; A2IDHmut—astrocytoma IDH mutant grade 2; A3IDHmut—astrocytoma IDH mutant grade 3; A4IDHmut—astrocytoma IDH mutant grade 4; Mut—mutant; WT—wild type.

**Table 2:** Characterization of the OSU cohort of grade 4 astrocytomas (N = 311 gliomas originally diagnosed as glioblastomas).

Sex	184 Male		122 Female		5 NA
Age (categorical)	156 < 60 years		149 ≥ 60 years		6 NA
Extension of surgery	263 Resection		42 Biopsy		6 NA
2021 WHO diagnosis	287 Glioblastoma		24 A4 IDHmut		
HMGB2 overexpression ^1^	76 High		223 Low		12 NA
Adjuvant treatment	110 RT + TMZ	41 RT only	38 None	112 Others ^2^	10 NA
Age (continuous)	25.8–91.2 years (Mean: 58.8; Median: 60.6)				
HMGB2 expression (score) ^3^	0–260 (Percentile 75 = 108)				
Overall survival	0.07–169.4 months (Mean: 15.9; Median: 10.5)				

^1^ Defined as a score above the 75th percentile (p75), this represents roughly 50% of tumor cells with moderate intensity or 33% of tumor cells with strong intensity. ^2^ Includes patients treated exclusively with TMZ and multi-drug treatments. ^3^ Calculated as percentage of positive cells multiplied by intensity of reaction. NA—not available; A4 IDHmut—astrocytoma grade 4 IDH mutant; RT—radiation therapy; TMZ—temozolomide.

**Table 3:** Multivariable survival analysis.

	coef	exp(coef)	se(coef)	z	*p*
IDH_IHCR132H	−0.0408	0.960013	0.545607	−0.075	0.94038
Geographic necrosis (yes)	−0.0727	0.929901	0.192487	−0.378	0.70575
Mitotic activity	0.01406	1.014155	0.005462	2.573	**0.01008**
Glomeruloid vasc (yes)	−0.0025	0.997489	0.185236	−0.014	0.98917
Sex (male)	0.06345	1.065509	0.176581	0.359	0.71934
Surgery (resection)	−0.2213	0.801442	0.312497	−0.708	0.47876
Adj treatment (others)	−2.6391	0.071428	0.377554	−6.99	**2.75 × 10^−12^**
Adj treatment (rt)	−1.4625	0.231664	0.323833	−4.516	**6.30 × 10^−6^**
Adj treatment (tmz + rt)	−2.5327	0.079442	0.301773	−8.393	**<2.00 × 10^−16^**
Age cat (>=60 years)	0.37144	1.449826	0.176933	2.099	**0.03579**
HMGB2Categ (score >= 108)	−0.6352	0.529813	0.206351	−3.078	**0.00208**

Bold highlights significant variables in the analysis.

## Data Availability

The data presented in this study are available on request from the corresponding author.

## Supplementary Materials

The following supporting information can be downloaded at: https://www.mdpi.com/article/10.3390/cancers16081516/s1. Figure S1: HMGB2 mRNA expression according to original diagnosis in TCGA datasets “Brain Lower Grade Gliomas” and “Glioblastoma multiforme”; Figure S2: Sankey plot of grades 2 and 3 diffuse gliomas (original diagnosis, column to the left) reclassified according to 2021 WHO criteria (column to the right), after evaluation of the current molecular criteria: IDH status, 1p19q alterations in chromosomes 7/10, TERT, EGFR, and/or CDKN2A/B [16]; Figure S3: Methylation probes with significant association with HMGB2 expression and overall survival; Figure S4: Regional heterogeneity analysis showed higher HMGB2 expression in the cellular tumor, compared with the infiltrating areas; Figure S5: Matrix of clinicopathologic correlations with HMGB2 expression; Table S1: Characterization of the discovery cohort submitted to liquid chromatography tandem mass spectrometry (LC-MS/MS) (N = 9); Table S2: Clinical characterization of the validation cohort 1 (N = 63); Table S3: Gene ontology (GO) background analysis of protein-coding genes in adult astrocytomas; Table S4: Gene ontology (GO) metanalysis HG versus LG areas; Table S5: Protein–protein interaction metanalysis; Table S6: Ingenuity Pathway Analysis (IPA) background analysis; Table S7: Ingenuity Pathway Analysis (IPA) of LG areas; Table S8: Ingenuity Pathway Analysis (IPA) of HG areas; Table S9: Methylation probes strongly associated with HMGB2 gene expression and overall survival (*p* ≤ 0.05; FDR ≤ 0.1).
