# Supplementary material for: Proteomic Analysis of Spatial Heterogeneity Identifies HMGB2 as Putative Biomarker of Tumor Progression in Adult-Type Diffuse Astrocytomas
Source: Cancers (Basel). 2024 Apr 16;16(8):1516. doi: 10.3390/cancers16081516 (PMC11049315; doi:10.3390/cancers16081516)
Supplement: Supplementary file 1 [file cancers-16-01516-s001.zip › Supplementary files HMGB2 final.pdf]

Supplementary Files - **Proteomic analysis of spatial heterogeneity identifies HMGB2 as putative biomarker of tumor progression in adult-type diffuse astrocytomas.**

Table S1: Characterization of the discovery cohort submitted to liquid chromatography tandem mass spectrometry (LC-MS/MS) (N=9)

Patient	Sample	Distance areas 1-2 (mm)	Distance areas 2-3 (mm)	Distance areas 1-3 (mm)	Origin of samples within the paraffin block
1	023	15	7	10	Multiple fragments
2	051	8	12	7	Single fragment
3	295	5			Multiple fragments
4	091	16			Multiple fragments
5	304	10	9	9	Single fragment
6	079	5	8	7	Single fragment
7	162	12	7	7	Multiple fragments
8	168	14	10	13	Multiple fragments
9	290	8	10	14	Multiple fragments

Table S2: Clinical characterization of the Validation cohort 1 (N=63)

**Sex**	44 Male	19 Female		
**IDH status**	54 Mutant	8 Wild type	1 NA	
**1p19q status**	30 Co-deleted	23 Intact	10 19q deleted	1 NA
**CDKN2A/B status**	6 Co-deleted	54 intact	3 NA	
**Extension of surgery**	45 Resection	15 Biopsy	3 NA	
**Tissue type**	37 Newly diagnosed tumors	23 Recurrent tumors	3 NA	
**Age**	21 – 75 years old (Mean: 42.7; Median: 41 years old)			
**Overall survival**	0.15 – 22.79 years (Mean: 5.9; Median: 3.27 years)			

Table S3: Gene Ontology (GO) Background analysis of protein-coding genes in adult astrocytomas

Table S4: Gene Ontology (GO) metanalysis HG versus LG areas

Table S5: Protein-protein interaction metanalysis

Table S6: Ingenuity Pathway Analysis (IPA) Background analysis

Table S7: Ingenuity Pathway Analysis (IPA) LG areas

Table S8: Ingenuity Pathway Analysis (IPA) HG areas

Table S9: Methylation probes strongly associated with HMGB2 gene expression and overall survival (p<=0.05; FDR<=0.1).

Name	Position	Location	Estimate	p_value	FDR	Survival Cox PH		Survival KM	
						HR	Raw p-value (FDR)	HR	Log rank p
cg03002428	174255734	TSS1500; TSS200; TSS1500	0.979	0.000988	0.01638				
cg00321931	174255552	TSS1500;1stExon TSS200; 5′UTR	0.86	0.00131	0.01638				
cg20409358	174255887	TSS1500; TSS1500; TSS1500	1.022	0.0117	0.0975				
**cg1937134***	174254281	Body; Body; Body	-0.198	0.0338	0.175	0.673	0.0203 (0.1319)	4.9 (1.0 – 23.2)	0.026
cg05944369	174255616	TSS1500; TSS200; TSS200	0.959	0.0404	0.175				
**cg21499459***	174254427	Body; Body; Body	-0.244	0.0502	0.175	0.548	0.0268 (0.1394)	0.218 (0.04 – 1.03)	0.035
**cg08269316***	174254507	Body; Body; Body	-0.216	0.0545	0.175	0.303	0.012 (0.104)	0.097 (0.01 – 0.76)	0.0062

***** - Methylation probes significantly related to overall survival

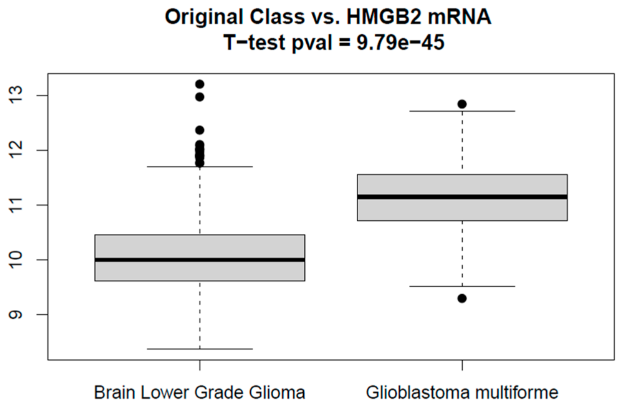

Figure S1: HMGB2 mRNA expression according to original diagnosis in TCGA datasets “Brain Lower Grade Gliomas” and “Glioblastoma multiforme”.

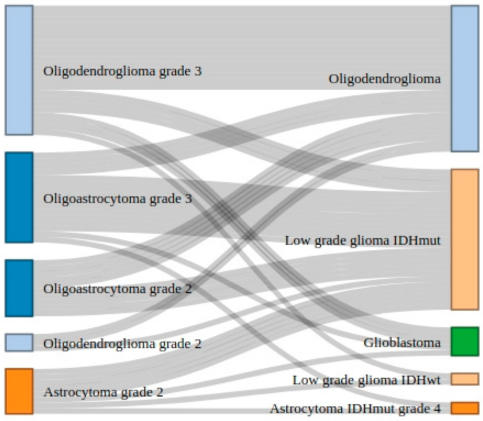

Figure S2: Sankey plot of grades 2 and 3 diffuse gliomas from validation cohort 1 (original diagnosis, column to the left) re-classified according to 2021 WHO criteria (column to the right), after evaluation of the current molecular criteria: IDH status, 1p19q alterations in chromosomes 7/10, TERT, EGFR and/or CDKN2A/B [15].

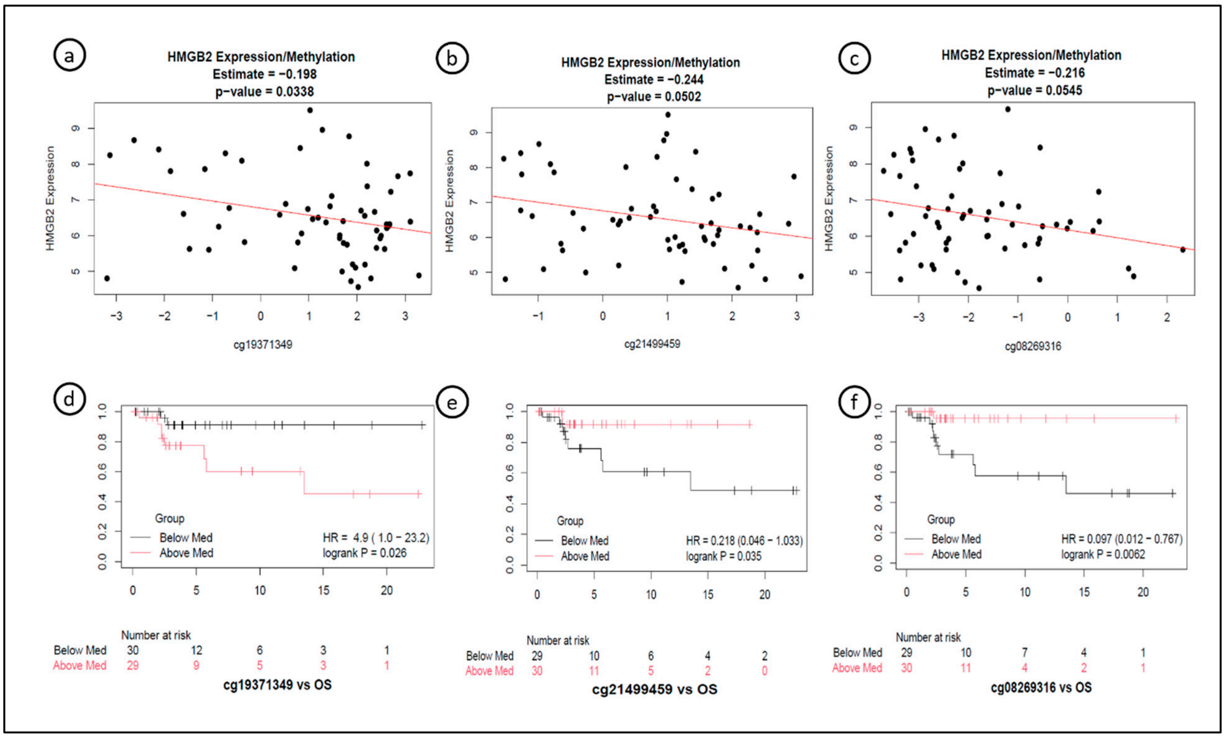

Figure S3: Methylation probes with significant association with HMGB2 expression and overall survival. The negative correlation between expression and methylation suggests that hypermethylation of the gene is associated with reduced expression, therefore better overall survival.

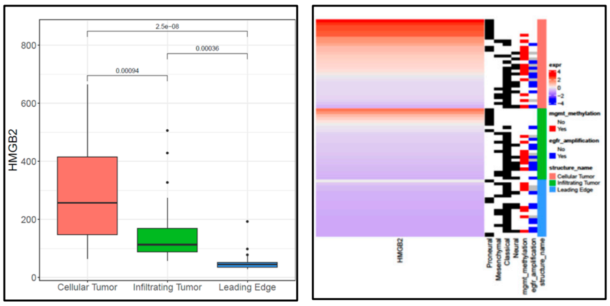

Figure S4: Regional heterogeneity analysis showed higher HMGB2 expression in the cellular tumor, compared with the infiltrating areas. The heatmap shows higher HMGB2 expression in Proneural, compared with Mesenchymal and Classical glioblastomas, suggesting some association with IDH status (Data from Ivy GAP).

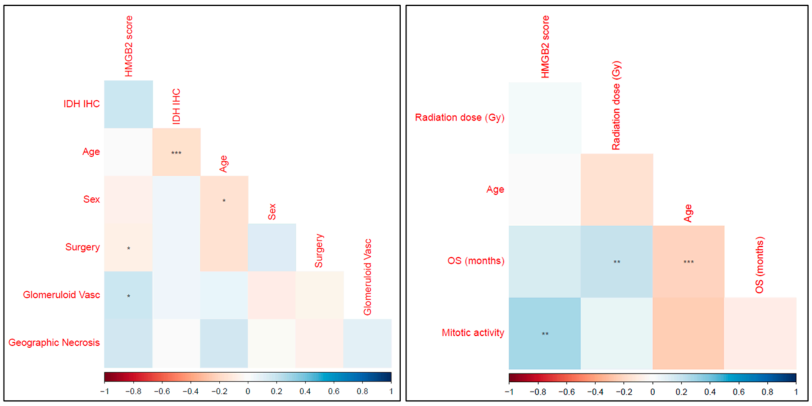

Figure S5: Matrix of clinicopathologic correlations with HMGB2 expression. (a) Spearman correlation of HMGB2 score with categorical variables. (b) Pearson correlation of HMGB2 score with continuous variables. HMGB expression was strongly associated with mitotic activity and glomeruloid blood vessels and trended to be associated with geographic necrosis and with IDH1 R132H. For both plots, *** = 0.001, ** = 0.01, * = 0.05 Negative correlation is shaded in red and positive correlation is shaded in blue.
